# Long Non-coding RNA Linc01224 Regulates Hypopharyngeal Squamous Cell Carcinoma Growth Through Interactions with miR-485-5p and IGF2BP3

**DOI:** 10.7150/jca.85019

**Published:** 2023-09-18

**Authors:** Lai Wei, Yuanhang Wu, Sisi Cai, Yulan Qin, Shuangchun Xing, Zhiqiang Wang

**Affiliations:** 1 Department of Otolaryngology, The Eighth Affiliated Hospital of Sun Yat-sen University, 518033, Shenzhen, China.; 2 Department of Otolaryngology, Affiliated Zhongshan Hospital of Dalian University, 116000, Dalian, China.; 3 Department of Oncology, The First Affiliated Hospital of Dalian Medical University, 116000, Dalian, China.; 4 Department of Otolaryngology, The First Affiliated Hospital of Dalian Medical University, 116000, Dalian, China.

**Keywords:** Hypopharyngeal Neoplasms, RNA, Long Noncoding, microRNAs, oncogenes, linc01224, miR-485-5p, IGF2BP3

## Abstract

Increasing evidence illustrates that long non-coding RNAs (lncRNAs) play significant oncogenic roles, including hypopharyngeal squamous cell carcinoma (HSCC). The function and mechanism of long non-coding RNAs (lncRNAs) in hypopharyngeal squamous cell carcinoma (HSCC) have not been fully elucidated. Therefore, this study aimed to investigate the role of a specific lncRNA, linc01224, in regulating the miR-485-5p/IGF2BP3 axis in HSCC. We confirmed the lncRNA expression profiles in 5 pairs of HSCC and normal tissues by lncRNA sequencing. Another 28 HSCC tissues were further validated by quantitative real-time PCR (qRT-PCR). qRT-PCR was also used to detect the expression levels of linc01224, miR-485-5p and IGF2BP3 in HSCC cell lines. Next, functional experiments *in vitro* and *in vivo* were applied to determine the effects of linc01224 silencing on tumor proliferation, migration, apoptosis and progression in HSCC. Linc01224 expression was significantly higher in HSCC tissues than in adjacent normal tissues. In addition, HSCC patients with low IGF2BP3 expression had good survival. *In vitro* assays were mechanistically performed to explore whether linc01224 positively regulates IGF2BP3 expression via its competitive inhibition of miR-485-5p. An *in vivo* animal model also confirmed that linc01224 could promote the occurrence and development of HSCC. Our study first identified that linc01224 plays an oncogenic role in HSCC. It suggests that linc01224 may act as a prognostic biomarker and potential therapeutic target for HSCC.

## Introduction

Hypopharyngeal squamous cell carcinoma (HSCC) accounts for approximately 0.5% of all tumors and 5% of head and neck tumors 1. In 2015, approximately 48100 new diagnoses of pharyngeal cancer and 22100 pharyngeal cancer-related deaths occurred in China 2. Despite the advancements in treatments such as surgery, chemotherapy, and radiation, patients with hypopharyngeal cancer are often diagnosed at an advanced stage, leading to a low 5-year survival rate of less than 30%. This is primarily attributed to delayed diagnosis, early metastasis, and a generally poor prognosis. 3. Therefore, it is essential to establish efficient prognostic biomarkers, therapeutic targets and molecular mechanisms for hypopharyngeal cancer 4.

Many studies have consistently indicated that the dysregulation of oncogenes and tumor suppressor genes plays a significant role in the initiation and progression of hypopharyngeal cancer. 5. Lin et al. reported that COL1A2 is upregulated in HSCC compared to normal controls; they also reported that a high COL1A2 expression level predicted a high locoregional recurrence rate and a less favorable disease-free survival rate 6. A recent study was observed by Kim EK et al. that FGFR1 expansion is an independent prognostic factor for hypopharyngeal and laryngeal squamous cell carcinoma 7. However, most of these studies contributed to protein-coding genes.

Increasing evidence indicates that long non-coding RNAs (lncRNAs) are essential in several pathophysiological processes of cancer 8-12. LncRNAs, defined as ncRNAs greater than 200 nt in length, exert their gene transcription regulatory function via epigenetic regulatory mechanisms 13. Various studies have also shown that abnormal expression of various lncRNAs is observed in hypopharyngeal cancer. Those lncRNAs are closely correlated with the cancer occurrence, diagnosis and prognosis 14-16. This study first detected long intergenic non-protein-coding RNA 1224 (linc01224) by RNA deep sequencing in 5 pairs of HSCC tissues and adjacent nontumor tissues. We found that linc01224 was highly overexpressed in HSCC and located on chromosome 19, with a length of 16842 nt. It is widely distributed in the duodenum, small intestine, colon and appendix in normal humans, and relevant studies have rarely been reported 17. Although a report has shown that linc01224 may play a vital role in breast cancer carcinogenesis to estimate the prognosis of patients with breast cancer, the molecular mechanisms of linc01224 remain primarily unclear 18.

The role of long non-coding RNAs (lncRNAs) - such as linc01224 - as competing endogenous RNAs (ceRNAs) is widely recognized in the scientific community. These ceRNAs have the ability to interact with mRNA or other lncRNA transcripts by competing for miRNA binding sites. This interaction plays a crucial role in modulating the expression of genes related to cancer 19. For example, the long non-coding RNA UCA1 promotes malignant phenotypes of prostate cancer by modulating the miR143/MYO6 axis as a ceRNA 20. In addition, linc01224 promotes cancer proliferation by sponging miR-485-5p to upregulate PAK4 in epithelial ovarian cancer 21. However, the function of the linc01224-miRNA-mRNA interaction in HSCC remains unclear.

In the present research, we hypothesized that linc01224 plays an essential role in the malignant progression of HSCC. To evaluate this hypothesis, we first assessed the expression of linc01224, which was upregulated, in HSCC clinical tissue specimens and cells. Then, we assessed the role and mechanism of linc01224 in HSCC cell apoptosis, proliferation, migration, and invasion both *in vitro* and *in vivo*. In addition, we computationally predicted what downstream RNAs could be bound by linc01224 and found that miR-485-5p could be bound explicitly by linc01224 and IGF2BP3 in StarBase v2.0 (http://starbase.sysu.edu.cn/). Moreover, knockdown of linc01224 decreased cell progression by regulating miR-485-5p. Consequently, the mechanisms underlying the effects of the linc01224/miR-485-5p/IGF2BP3 pathway were confirmed.

## Materials and Methods

### Clinical tissue specimens

Twenty-eight pairs of HSCC tissues and matched normal tissues were collected between February 2014 and December 2018 from our hospital. There were no HSCC patients who received preoperative treatments, radiotherapy or chemotherapy, and two histopathologists proved the tissue sample diagnoses. The patient characteristics of these patients are shown in Table 1. All tissue specimens were collected during surgery and frozen in liquid nitrogen at -80°C for further analysis.

### RNA sequencing data analysis

RNA sequencing was performed with the Illumina TruSeq^®^ Stranded RNA LT Kit. Briefly, 0.1-1 µg of total RNA from each sample was used for library preparation, including removing ribosomal RNA (rRNA), synthesizing cDNA, enrichment of DNA fragments, and sequencing the library. Sequence alignment was confirmed using the Burrows-Wheeler aligner (BWA). Each sample's resulting single BAM file was passed through a quality control pipeline embedded in the OncoDecoder^TM^ system (Genomic Future, Inc.). OncoDecoder^TM^ has integrative workflows that contain a set of tools for analyzing next-generation sequencing data, including tumor and matched normal pairs; OncoDecoder^TM^ performs quality control, alignment, local realignment, mutation calling, small insertion and deletion identification, copy number variation analysis, and coverage calculations, among other functions 22.

### Cell culture

The human HSCC FaDu cell line was acquired from Procell (Wuhan, China). FaDu cells were cultured in Dulbecco's modified Eagle's medium (DMEM; Gibco, Grand Island, NY, USA) supplemented with 10% fetal bovine serum (FBS; Logan City, UT, USA). Cells were preserved in a humidified incubator (37°C, 5% CO2).

### RNA extraction and quantitative real-time PCR analysis

Total RNA from HSCC cells was obtained using TRIpure reagent (BioTeke, Beijing, China) according to the manufacturer's protocol. A NanoDrop ND-2000 spectrophotometer (Thermo Scientific, Wilmington, DE, USA) was applied to determine RNA quantity and quality. RNA was reverse transcribed into cDNA with the Reverse Transcription Kit (BioTeke). Quantitative real-time PCR (qRT-PCR) was performed with SYBR Green (Solarbio, Beijing, China) and an Exicycler 96 system (BIONEER, Daejeon, South Korea). The levels of linc01224 and IGF2BP3 expression were normalized to that of β-actin. Samples were analyzed in triplicate. The primers of forward and reverse sequences were as follows: linc01224, 5'-GAGCCAGGCACCCGTTTA-3' and 5'-GGTTGACAAGTGGTGGAATCTG-3'; IGF2BP3: 5'-TCGGAACATCACCAAACA-3' and 5'-GGTGCCTTCAGGAGTAGAG-3'; and β-actin: 5'-CTTAGTTGCGTTACACCCTTTCTTG-3' and 5'-CTGTCACCTTCACCGTTCCAGTTT-3'. The relative expression of linc01224 was calculated by the 2^-△△CT^ method.

### Cell transfection

Three shRNAs against linc01224 and a negative control group were engineered and synthesized by Wanleibio (Shenyang, China). The primer sequences of the three different shRNAs were as follows: shRNA-1, GCACAGACAGCTAAGATG; shRNA-2, GATCAAAGGCGCCTGTAAT; and shRNA-3, GCAGGAGTCATTGTCACAT. The most efficient knockout group was confirmed by qRT-PCR and then used to create mature cell lines. FaDu cells were transplanted into 6-well plates and transfected with specific shRNA and pRNA-H1.1 vector (GenScript Biotech, Nanjing, China) using Lipofectamine 2000 (Invitrogen, Carlsbad, CA, USA). MiR-485-5p mimics, miR-485-5p mimics NC (mimics NC), miR-485-5p inhibitors, and miR-485-5p inhibitors NC (inhibitors NC) were performed from RiboBio (Guangzhou, China). For two weeks, the transfected cells were treated with G418 (300 µg/ml).

### Cell Counting Kit-8 (CCK-8) assay

The CCK-8 (Wanleibio, China) assay evaluated the proliferation of FaDu cells following the manufacturer's instructions. Four × 10^3^ cells were seeded in quintuplicate into a 96-well plate. At 24, 48, 72, and 96 hours after seeding, cell viability was measured using the CCK-8 assay. Specifically, 10 μL of CCK-8 reagent was added to each well at the designated time points. A microplate reader detected the absorbance was detected at 450 nm using a microplate reader (ELX-800, BIOTEK, USA).

### Cell apoptosis analysis

Cells were extracted and washed twice with phosphate-buffered saline (PBS). Then those cells were coated in 500 µl of binding buffer containing 5 µl of Annexin V-Light 650 and 5 µl of propidium iodide (PI; Wanleibio) at room temperature in the dark for 15 min. Flow cytometry with a NovoCyte cytometer (ACEA Biosciences, San Diego, CA, USA) was performed to detect the stained cells.

### Cell cycle analysis

Cells were transfected for 24 h. Then, cells were harvested, washed twice with PBS and fixed with ice-cold 70% ethanol at four °C for two h. Cells were incubated with RNase (Wanleibio) at 37°C in the dark for 30 min and then incubated with PI at four °C in the dark for 30 min. Flow cytometry (BD Biosciences, San Diego, CA, USA) was employed to determine cell cycle distribution.

### RNA FISH

According to the manufacturer's instructions, RNA FISH was performed using fluorescent *in situ* hybridization kit (210716, GenePharma, China). Cy3-labeled probes for linc01224 and Dig-labeled miR-485-5p probes were synthesized by Wanlei Biotechnology Co. Ltd (China). Images were recorded with a confocal laser scanning microscope (AF6000, Leica, Germany).

### Luciferase reporter assay

The sequences of linc01224 and IGF2BP3-3'UTR, along with their corresponding mutations, were synthesized, designed, and inserted into the luciferase reporter vector GP-miRGLO (Genepharma, China). These constructs were denoted as linc01224-WT, linc01224-MUT, IGF2BP3-3'UTR-WT, and IGF2BP3-3'UTR-MUT, respectively. The plasmids were co-transfected with miR-485-5p mimics or inhibitors into 293T cells. Then, the relative luciferase activity was measured using a Dual-Luciferase Assay Kit (Promega, USA) according to the manufacturer's protocol.

### Cell migration and invasion assays

We used Transwell chambers (Corning, Tewksbury, MA, USA) to measure the migration capacity of FaDu cells. A total of 5 × 10^3^ cells in 200 µl of serum-free medium were placed into the upper chamber. Then, 800 µl of a medium comprising 20% FBS was introduced to the bottom chamber. After incubation for 24 h, cells in the lower chamber were fixed with 4% paraformaldehyde and stained with 0.5% crystal violet. The design of the cell invasion assay was similar to that of the cell migration assay, and Transwell membranes were coated with Matrigel (BD Biosciences). We used an inverted microscope (magnification 200x) to count the numbers of cells that migrated or invaded in five random fields.

### Western blot analysis

The manufacturer's protocol extracted total protein from cell lines with a Total Protein Extraction Kit (Wanleibio). Then, 40 µg of total protein was separated by 10% SDS-PAGE and transported to polyvinylidene difluoride (PVDF) membranes (Millipore, MA, USA). The PVDF membrane was blocked with 5% skim milk at room temperature for one h. After that, the membranes were incubated with primary antibodies against IGF2BP3 (Proteintech Group, Wuhan, China) and β-actin (Wanleibio) at four °C overnight; HRP-conjugated goat anti-rabbit IgG antibody (1:5000; Wanleibio) was incubated for 45 min at 37°C. The protein bands were visualized with a gel imaging system (LiuYi, Beijing, China).

### Tumor xenograft experiments

FaDu cells (1 x 10^7^) transduced with linc01224 or shRNA-linc01224 were dissolved in 0.1 ml PBS and injected into the flanks of 5-week-old male BALB/c athymic nude mice obtained from Dalian Medical University. Tumor volumes were calculated using hand calipers every week after the injection. At 30 days, mice were sacrificed, and tumor volumes and weights were recorded. Tumors were evaluated using hematoxylin and eosin (H&E) staining and immunohistochemistry. The animal experiments were performed by the Animal Care and Use Committee of The Affiliated Zhongshan Hospital of Dalian University.

### Statistical analysis

The statistical analyses were performed by SPSS 20.0 software (SPSS, Chicago, IL, USA). These results are shown as the mean ± SD (standard deviation) of three independent experiments. The student's t-test was used to compare data between control and transfected cells, and the Kaplan-Meier method was used to evaluate survival. Graphs generated using GraphPad Prism 6.0 Software (GraphPad Inc., San Diego, CA, USA). P values of <0.05 were considered to be statistically significant.

## Results

### lncRNA linc01224 is overexpressed in HSCC

**Figure 1:**
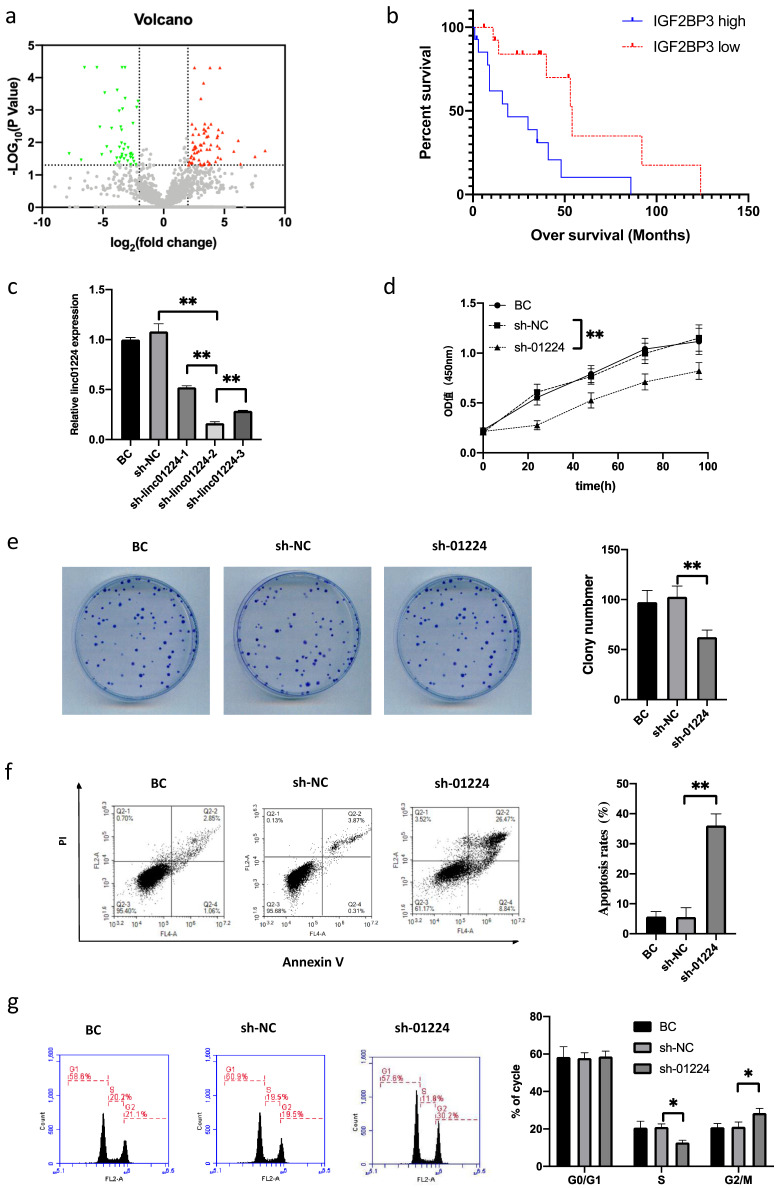
**Volcano plot of the differences in 154 differentially expressed lncRNA expression profiles between HSCC tissues and adjacent non-tumor tissues. a** High expression (red) and low expression (blue) correlations are shown. Gray dots show the lncRNAs with expression of log2(fold change)<2. IGF2BP3 expression correlates with HSCC progression.** b** Kaplan-Meier curves of the survival of 28 patients were evaluated using the log-rank test. Linc01224 knockdown markedly suppressed the proliferation and arrested the cell cycle of FaDu cells *in vitro***. c** shRNA knockdown of linc01224 expression. ShRNA dramatically suppressed linc01224 expression at the RNA level compared with that in control cells, as detected by qRT-PCR. **d** CCK-8 assays were used to detect the proliferation of FaDu cells. **e** A cell clone formation assay was performed to measure the clonogenicity of FaDu cells. **f** Annexin V/PI staining and flow cytometry were performed to assess apoptosis in FaDu cells after shRNA transfection. **g** The cell cycle was assessed by flow cytometry after PI staining of linc01224-downregulated FaDu cells. ^*^P<0.05, ^**^ P<0.01.

To identify lncRNAs with differential expression in head and neck squamous cell carcinoma (HSCC), we analyzed RNA sequencing data comparing the expression profiles of mRNAs and lncRNAs between 5 HSCC tumor tissues and matched adjacent nontumor tissues from HSCC patients. A total of 154 lncRNAs were differentially expressed in HSCCs compared to adjacent normal tissues, of which 68 were upregulated and 86 were downregulated (Fig. 1a). We identified a novel lncRNA, linc01224 (also known as CTB-175P5.4), that exhibited a 4.28-fold higher expression in HSCC tumor tissues compared to matched adjacent nontumor tissues. This significant upregulation of linc01224 expression in HSCC tissues caught our attention.

### High levels of IGF2BP3 correlate with poor prognosis in HSCC patients

To investigate the clinical significance of IGF2BP3 in HSCC, we performed immunohistochemical staining to analyze IGF2BP3 expression levels in 28 HSCC samples. The HSCC patients were stratified into low and high IGF2BP3 expression subgroups, using the cohort's median IGF2BP3 expression as the cut-off. As summarized in Table 1, correlation analysis between IGF2BP3 expression and clinicopathologic characteristics of the 28 HSCC patients showed that high IGF2BP3 expression did not correlate with tumor size (P>0.05), tumor grade (P>0.05), or tumor-node-metastasis (TNM) stage (P>0.05). This indicates that IGF2BP3 expression levels do not associate with these major clinicopathologic features in the HSCC cases examined. We further assessed the association between IGF2BP3 and the survival of 28 HSCC patients with follow-up data. Importantly, Kaplan-Meier survival analysis showed that HSCC patients with high IGF2BP3 expression had significantly poorer overall survival than those with low IGF2BP3 expression (P<0.05, Fig. 1b). Collectively, these results indicated that the upregulation of IGF2BP3 might play an essential role in HSCC progression.

### Knockdown of linc01224 inhibits FaDu cell proliferation and induces cell cycle arrest

Linc01224 expression is positively correlated with IGF2BP3 expression. linc01224 was also upregulated in HSCC tissues, so we next investigated the effects of linc01224 downregulation on FaDu cells.

First, we confirmed the expression level of linc01224 in FaDu cells by qRT-PCR. Consistent with our previous experimental data using ten pairs of HSCC tumor tissues and adjacent nonneoplastic tissues by transcriptome sequencing, the expression of linc01224 was upregulated in FaDu cells, which may mean that linc01224 can participate in the pathogenesis of HSCC.

Then, to discover the functional role of linc01224 in FaDu cells, we performed loss-of-function experiments *in vitro*. When linc01224-shRNAs (sh-linc01224-1, sh-linc01224-2 and sh-linc01224-3) were targeted, the expression of linc01224 was downregulated in FaDu cells compared with negative control FaDu cells (shRNA-NC) and blank control (BC) FaDu cells by qRT-PCR. Next, sh-linc01224-2 (sh-01224) was retained for further experiments, as it had the highest knockdown efficiency among the three shRNAs (Fig. 1c).

To assess the functional role of linc01224 in regulating cell proliferation, we performed CCK-8 and colony formation assays after linc01224 knockdown. The CCK-8 results showed that linc01224 knockdown significantly inhibited cell proliferation (Fig. 1d). Similarly, the colony formation assay demonstrated that linc01224 downregulation led to markedly decreased colony numbers (Fig. 1e). Together, these results reveal that knockdown of linc01224 suppresses the proliferative capacity of cells. In addition, the percentage of early apoptotic cells was significantly increased in the sh-linc01224 groups compared to the control groups (Fig. 1f). Moreover, significant S and G2/M cell cycle arrest was observed in linc01224-silenced cells (Fig. 1g).

**Figure 2:**
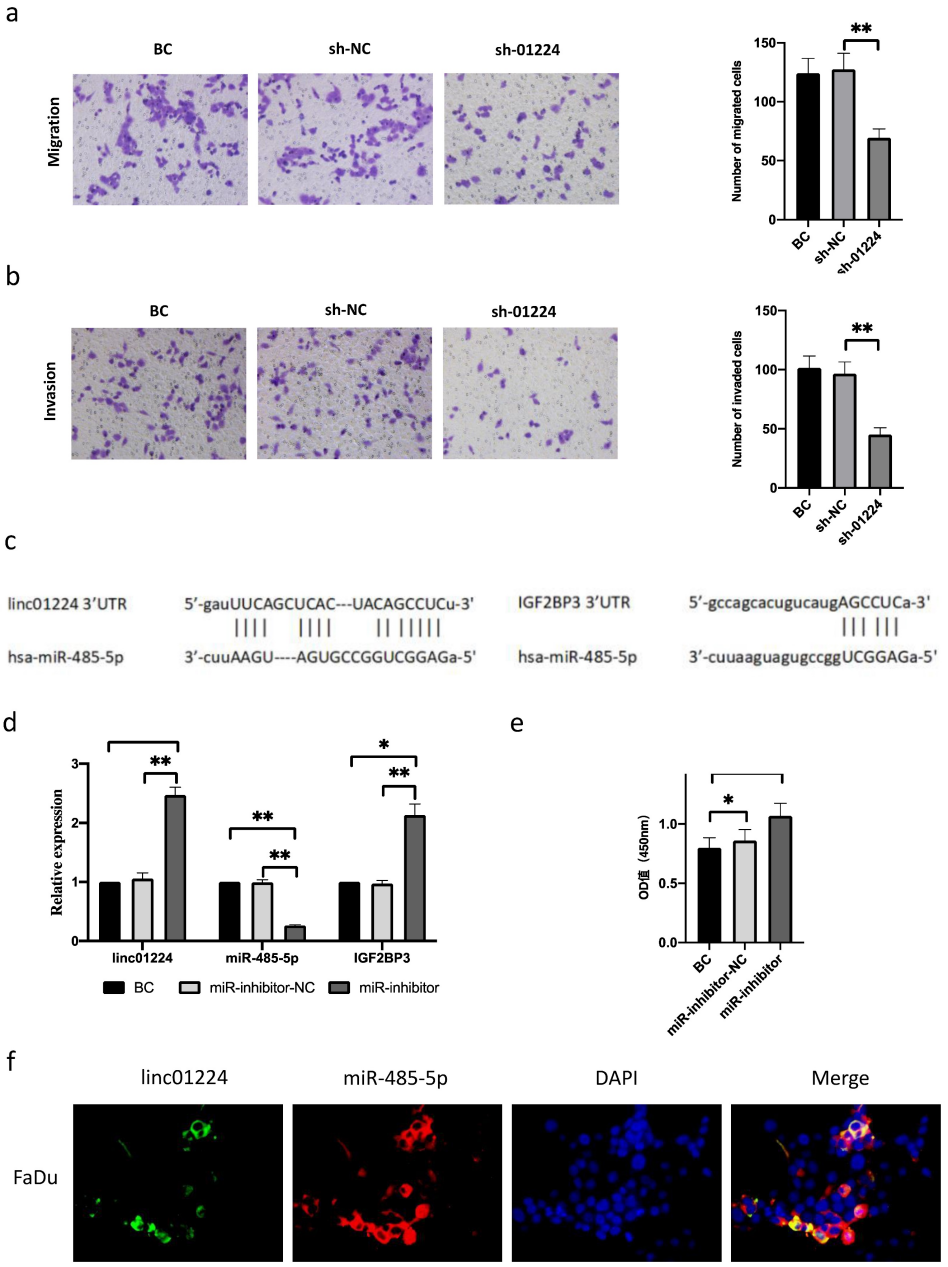
**Linc01224 silencing inhibits FaDu cell migration and invasion. a** Transwell assays were used to preformed the migratory capacity of FaDu cells transfected with control cells or linc01224 shRNA. **b** Transwell assays were used to survey the invasive ability of FaDu cells transfected with NC or linc01224 shRNA. MiR-485-5p is predicted to interact with linc01224. **c** Predicted miR-485-5p sites in the linc01224 and IGF2BP3 sequences. **d** Linc01224 and IGF2BP3 expression were increased after miR-485-5p inhibitor transfection. **e** The OD value of linc01224 was increased after miR-485-5p inhibitor transfection. **f** The FISH assay showed that linc01224 was predominantly localized in the cytoplasm. Nuclei were stained with DAPI blue, cytoplasmic linc01224 was stained green, and cytoplasmic linc01224 was stained red. (magnification, 400×). ^*^P<0.05, ^**^ P<0.01.

In addition, we observed that cell invasion and migration were suppressed in FaDu cells transfected with sh-linc01224 compared with cells transfected with sh-NC using a Transwell assay (Fig. 2a-b). Moreover, western blot analysis revealed that the expression of IGF2BP3 protein was decreased, which further proved the suppressive effect of linc01224 silencing on cell migration (Fig. 5c).

### MiR-485-5p is a target of linc01224

To explore the potential mechanisms of linc01224 functions in HSCC, we predicted miRNA-containing binding sites of linc01224 and IGF2BP3 using StarBase v2.0. The results showed that both linc01224 and IGF2BP3 contained predicted miR-485-5p targeting sites (Fig. 2c).

Then, FaDu cells were transfected with miR-485-5p inhibitors, and miR-485 knockdown was confirmed by real-time PCR (Fig. 2d). We observed a significant increase in linc01224 and IGF2BP3 expression compared with that in the control group, as measured by real-time PCR (Fig. 2e), suggesting that linc01224 and IGF2BP3 expression is significantly elevated when the miR-485-5p level is modulated in FaDu cells.

The results of the FISH assay also revealed that linc01224 and miR-485-5p were predominantly localized in the cytoplasm (Fig. 2f). These results also indicated that linc01224 and miR-485-5p might function in the cytoplasm.

### Knockdown of miR-485-5p promotes FaDu cell migration and invasion and modulates the cell cycle

**Figure 3:**
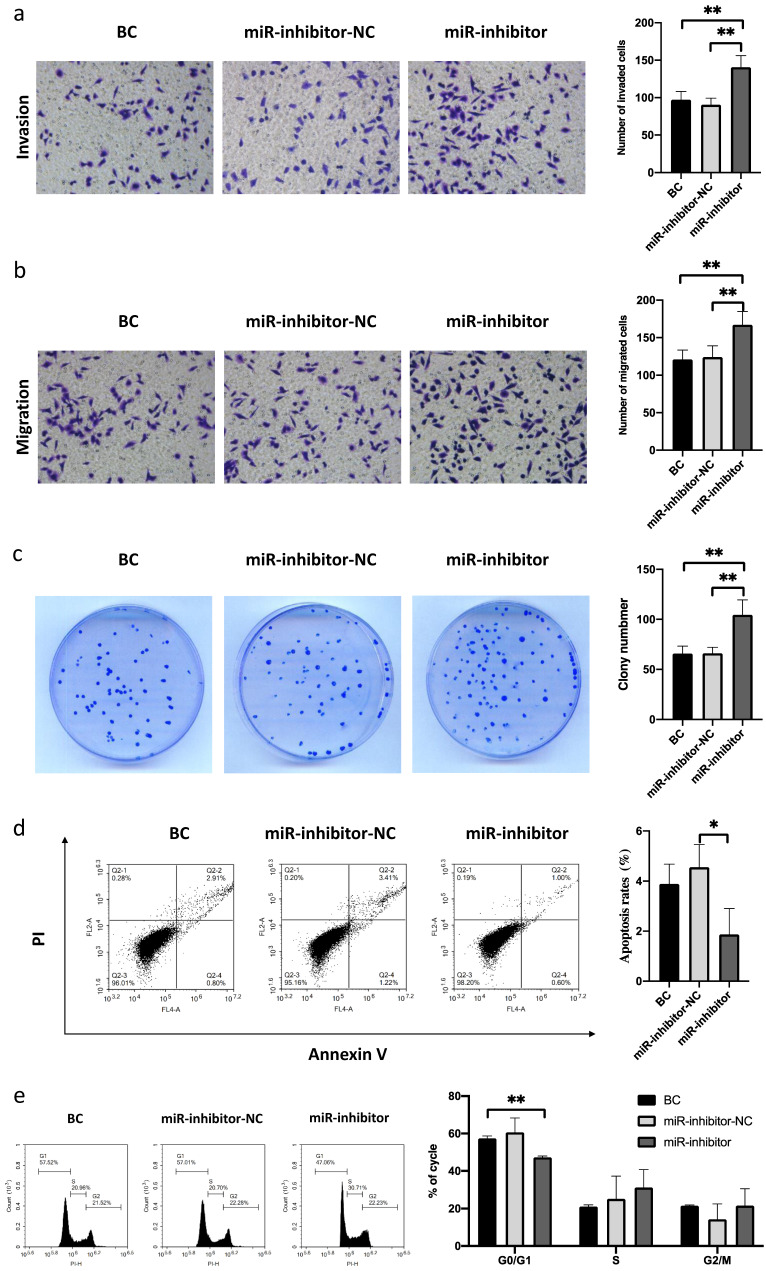
**MiR-485-5p silencing promoted FaDu cell growth, migration, and invasion and induced cell cycle arrest. a-b** miR-485-5p silencing promoted the migration and invasion of FaDu cells. **c** The number of FaDu cell colonies was increased after miR-485-5p knockdown. **d** Flow cytometry assay revealed that the percentage of apoptotic HSCC cells was reduced by miR-485-5p knockdown. **e** MiR-485-5p knockdown led to S and G2/M arrest in FaDu cells. ^*^P<0.05, ^**^ P<0.01.

Next, we attempted to confirm the roles of miR-485-5p in the migration and invasion of FaDu cells. The results showed that the migration and invasion abilities of FaDu cells were markedly enhanced by miR-485-5p silencing (Fig. 3a-e). Additionally, Western blot analysis revealed that miR-485-5p silencing increased IGF2BP3 expression in FaDu cells (Fig. 5c). Therefore, we determined that miR-485-5p silencing can promote the migration and invasion of FaDu cells.

### Silencing of miR-485-5p reverses the inhibitory effects of sh-linc01224 on FaDu cells

**Figure 4:**
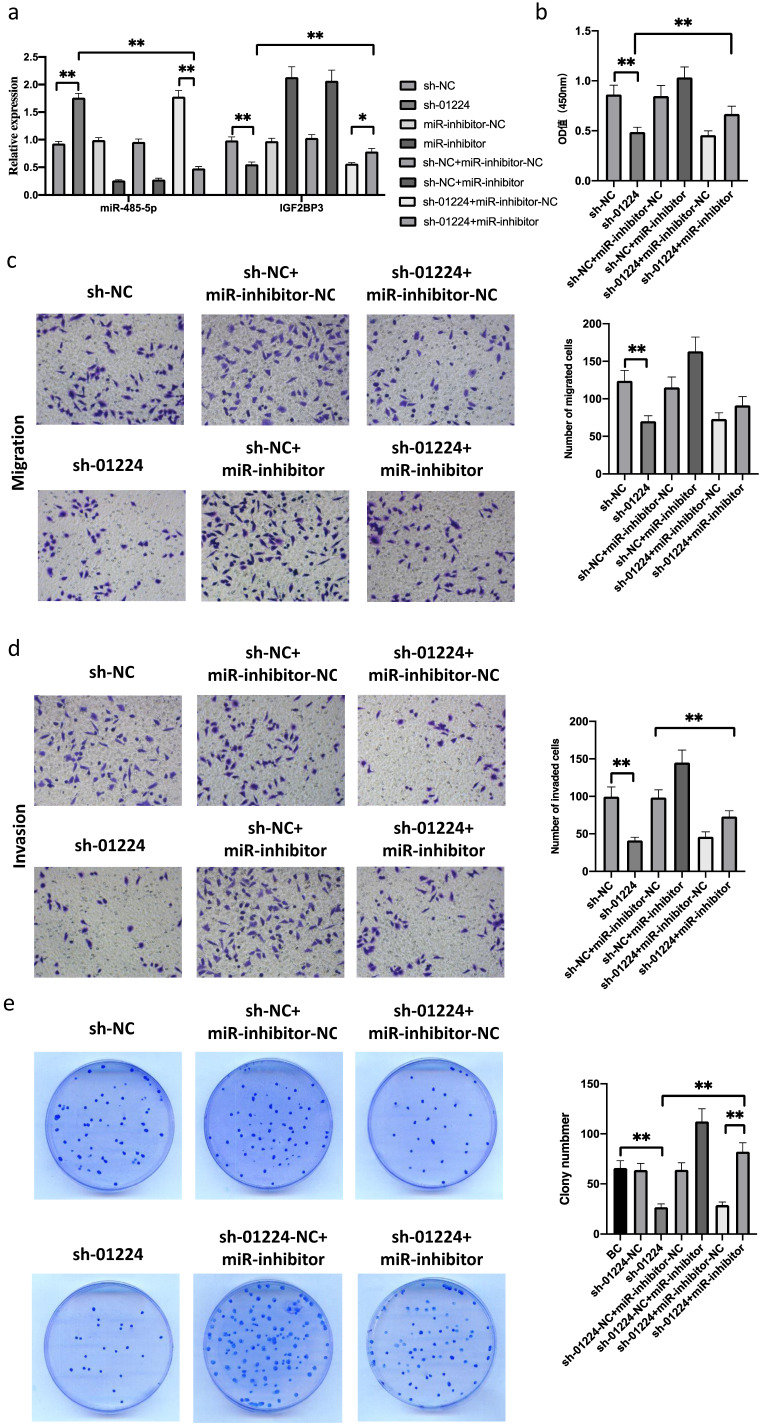
**MiR485-5p inhibitor reversed the function of sh-linc01224 in FaDu cells *in vitro***. a sh-linc01224 dramatically suppressed linc01224 expression and restrained IGF2BP3 expression at the RNA level compared with control cells by qRT-PCR. b CCK-8 assay showed that the introduction of miR-485-5p inhibitor restrained the proliferation-attenuating effect of sh-linc01224 in FaDu cells. c The abilities of cell invasion and migration induced by linc01224 were significantly promoted after introducing miR-485-5p inhibitor in FaDu cells, and miR-485-5p inhibitor reversed the inhibitory effect of sh-linc01224 on FaDu cell invasion, as measured by a Transwell assay. d The introduction of miR-485-5p inhibitor suppressed the colony formation-attenuating effect of sh-linc01224 in FaDu cells. ^*^P<0.05, ^**^ P<0.01.

To elucidate the mechanisms involved in the role of linc01224 in HSCC, we investigated the expression of IGF2BP3 and miR-485-5p in FaDu cells following linc01224 knockdown. The expression of miR-485-5p in FaDu cells with linc01224 knockdown was significantly higher than that in control cells. In contrast, the expression of IGF2BP3 in FaDu cells with linc01224 knockdown was markedly lower than that in the control cells (Fig. 4a).

**Figure 5:**
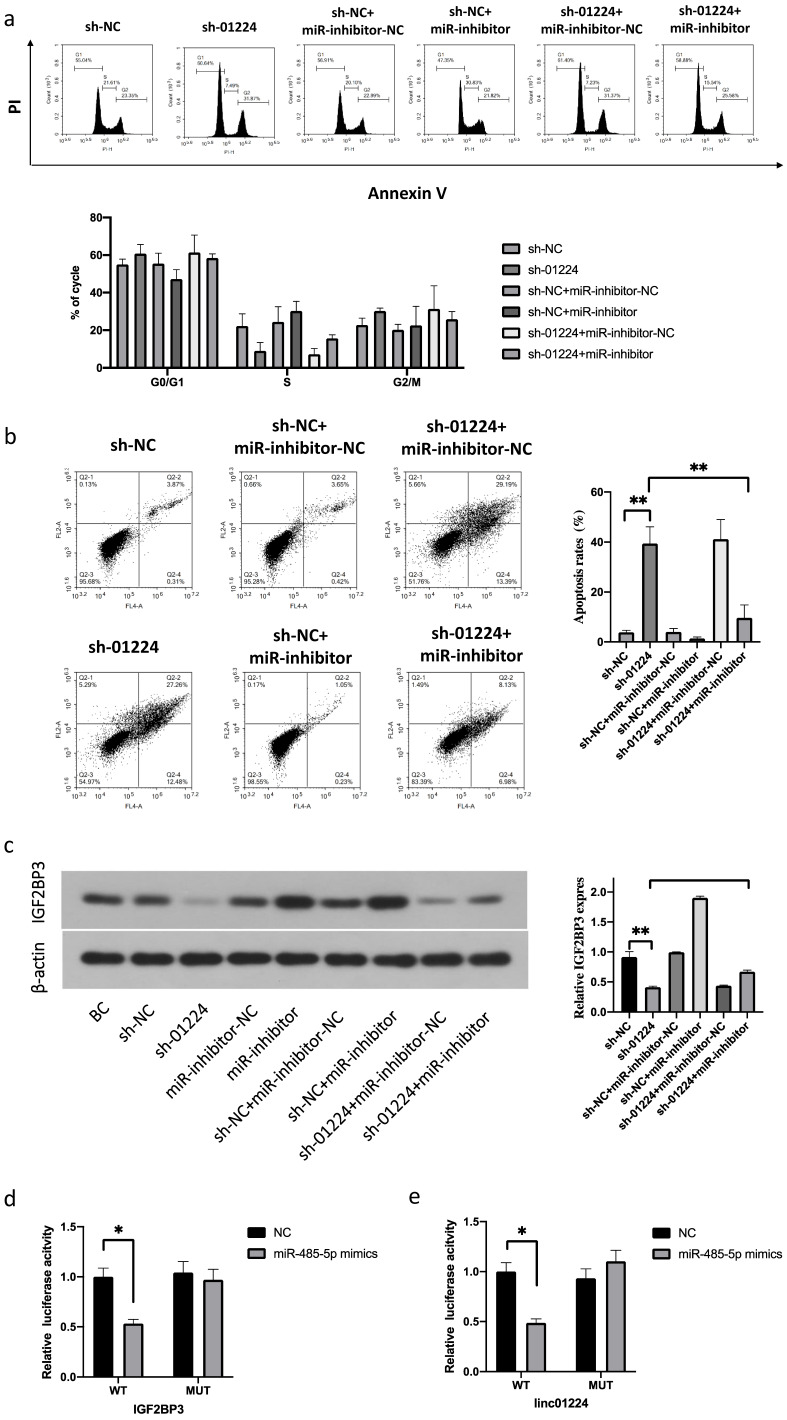
**FaDu cells with linc01224 knockdown were transfected with miR-485-5p inhibitor. a** Cell cycle assay. **b** Apoptosis assays were performed to measure the cell cycle and apoptosis progression. Western blot experiments were used to detect IGF2BP3 expression in FaDu cells transfected with control linc01224 shRNA.** c** miR-485-5p downregulation increased the level of IGF2BP3, and this effect was reversed by linc01224 downregulation in FaDu cells, as detected by Western blotting. **d** Luciferase activity in 293T cells co-transfected with the reporter plasmid inserted with the wild-type or mutated IGF2BP3 sequences and miR-485-5p mimics or mimic-NC.** e** Luciferase activity in 293T cells co-transfected with the reporter plasmid inserted with the wild-type or mutated linc01224 sequences and miR-485-5p mimics or mimic-NC. ^*^P<0.05, ^**^ P<0.01.

Next, to investigate whether linc01224 suppresses FaDu cell proliferation, migration and invasion by targeting miR-485-5p, we simultaneously downregulated linc01224 in FaDu cells and transfected them with a miR-485-5p inhibitor. Notably, the silencing of miR-485-5p significantly reversed the inhibitory effect of sh-linc01224 on FaDu cells (Fig. 4b-e, 5). This result indicated that miR-485-5p suppresses linc01224 function.

We performed luciferase reporter assays to verify whether miR-485-5p could bind to the 3′-UTRs of IGF2BP3 and linc01224. Luciferase activity was significantly decreased in miR-485-5p mimic-transfected cells but was increased in miR-485-5p inhibitor-transfected cells (Fig. 5d). Furthermore, the point mutations of the miR-485-5p targeting site were able to abolish the overserved effects of miR-485-5p on the 3′-UTR of IGF2BP3 and linc01224 (Fig. 5e).

### Linc01224 knockdown inhibits tumorigenesis of HSCC *in vivo*

**Figure 6:**
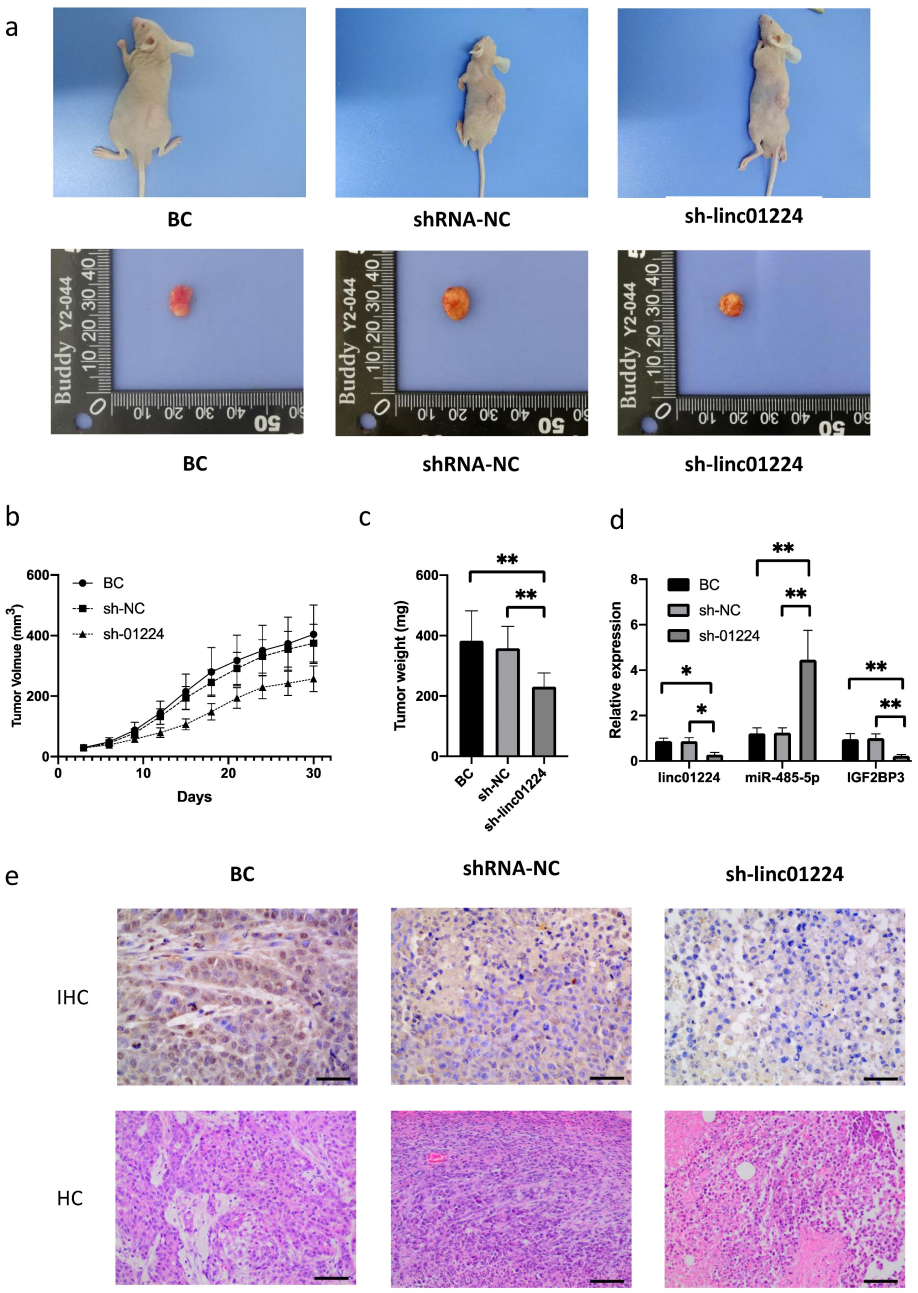
**Linc01224 knockdown significantly suppressed HSCC tumor growth *in vivo*.** Nude mice were subcutaneously injected with FaDu cells stably transfected with either linc01224 shRNA or control shRNA. **a** The analysis of the tumor growth curve demonstrated that the suppression of linc01224 resulted in the hinderance of HSCC growth in mice. **b** On the 30th day following implantation, the subcutaneous tumors were collected and their weights were measured. **c** The xenograft tissues were analyzed using qRT-PCR to determine the expression levels of linc01224, miR-485-5p, and IGF2BP3. **d** H&E and immunostaining of IGF2BP3 in xenograft tissues arise from the linc01224 knockdown and control groups. ^*^P<0.05, ^**^ P<0.01.

To further validate the biological roles of linc01224 in HSCC tumorigenesis *in vivo*, FaDu cells with linc01224 knockdown were subcutaneously injected into nude mice. Tumor growth curves and tumor weight assessment demonstrated that linc01224 knockdown significantly suppressed tumor growth in mice (P < 0.05). Tumor tissues were extracted for qRT-PCR analysis of linc01224, miR-485-5p and IGF2BP3. We confirmed that lower expression of linc01224 was detected in tumor tissues derived from linc01224 knockdown cells than control cells (P < 0.05, Fig. 6). The results of immunostaining analysis indicated that the expression of IGF2BP3 was significantly downregulated in FaDu cells transfected with sh-linc01224 compared to those in the control group. Taken together, this result suggests that linc01224 knockdown suppresses HSCC tumorigenesis *in vivo*.

## Discussion

We first confirmed that linc01224 is upregulated in HSCC tissues and cells compared to normal controls. High linc01224 expression was demonstrated to regulate IGF2BP3, positively associated with poor survival in HSCC patients. Furthermore, the knockdown of linc01224 exhibited a substantial decrease in the migratory, proliferative, and invasive capacities of FaDu cells when evaluated *in vitro*. Moreover, this knockdown also demonstrated a remarkable inhibition of tumor growth *in vivo*. Furthermore, silencing of miR-485-5p reversed the inhibitory effects of linc01224 downregulation. These results were same as those of previous reports and indicated that linc01224 functions as an oncogene in HSCC 17,21.

Hundreds of lncRNAs are abnormally processed by RNA sequencing in several human cancers, suggesting that lncRNAs are critical regulators in stimulating tumorigenesis 23. Some of these lncRNAs have been well distinguished, and their potential mechanisms in cancer cells have been unconcealed; these biological actions include regulation of proliferation, invasion, metastasis and progression. For instance, DLEU2 is a poor prognostic factor for laryngeal squamous cell carcinomas, and its miR-30c-5p/PIK3CD/Akt axis may represent a valuable therapeutic target 24. It has been reported that PMS2L2 may downregulate miR-25 expression to inhibit gastric adenocarcinoma 12. However, while an increasing number of lncRNAs have been identified as vital biological molecules in carcinogenesis, only a few HSCC-related lncRNAs are well recognized.

In this study, linc01224 was markedly upregulated in 5 HSCC tissues compared to adjacent nontumor tissues and caught our attention. Growing evidence has shown that linc01224 plays a vital role in tumor progression. For instance, upregulated linc01224 was related to advanced tumor stages and poor survival in hepatocellular carcinoma 17. Similarly, in this study, we found that the expression of linc01224, a cancer-promoting gene, was markedly enhanced in HSCC tissues by RNA sequencing. Next, qRT-PCR analysis validated the expression levels of linc01224 in HSCC tissue samples and the FaDu cell line. The qRT-PCR results revealed significantly higher linc01224 expression in HSCC tissues compared to matched non-tumor tissues, as well as in FaDu cells. These findings suggest that linc01224 may play a role in HSCC tumorigenesis or progression. Thus, we investigated the effect of linc01224 in FaDu cells by stably knocking down linc01224. Both *in vitro* and *in vivo* experiments showed that linc01224 silencing inhibited cell proliferation, migration and invasion capacities and promoted apoptosis. These results indicate a tumor suppressor role of linc01224 in HSCC and highlight the need for further study of the molecular mechanism of linc01224 in HSCC.

To date, it is reported that lncRNAs have an active role in regulating sequence complementarity with miRNAs as sponges. For example, Li X et al. reported that the long non-coding RNA DLEU2, significantly upregulated in laryngeal squamous cell carcinoma, encourages tumor growth and metastasis and triggers the PIK3CD/Akt axis by blocking the expression of miR-30c-5p 24. In addition, lncRNA THAP9-AS1 has been proven to act as a miR-484 sponge and provides a therapeutic target for patients with pancreatic ductal adenocarcinoma 25. In parallel, as a marker associated with gallbladder cancer, lncRNA PVT1 has been reported to induce invasion, metastasis and apoptosis resistance of gallbladder cancer cells by silencing HK2 via upregulation of miR-143^26^. Unfortunately, the expression and signaling mechanism of linc01224 as a ceRNA in HSCC remains unclear.

To identify the underlying molecular mechanism of linc01224 activity in FaDu cells, bioinformatics analysis was performed. This analysis revealed that miR-485-5p has known tumor suppressor roles, its interaction with linc01224 may be important for understanding linc01224's molecular functions in FaDu cells. MiR-485-5p also has been demonstrated to act as a tumor suppressor in several cancer types including breast cancer 27 and ovarian cancer 28, contained potential binding sites for linc01224 21. In the present study, our study revealed that the downregulation of linc01224 is related to the upregulation of miR-485-5p in FaDu cells. This was also confirmed by Xing, S et al. in epithelial ovarian cancer 21. Thus, these data indicate that linc01224 plays an oncogenic role in HSCC by downregulating miR-485-5p expression. In this study, RNA FISH showed that linc01224 and miR-485-5p colocalized in the cytoplasm of FaDu cells. Furthermore, a dual-luciferase reporter assay proved a direct interaction between linc01224 and miR-485-5p. In addition, to examine the association between linc01224 and miR-485-5p, we first described that suppression of miR-485-5p reversed the suppressive effects of linc01224 downregulation on the proliferation, migration and invasion of FaDu cells.

MicroRNAs are short (20-24 nt) non-coding RNAs that have been documented as inhibitors of malignant tumors 29-31. MiR-485-5p is a functional miRNA that has received much attention in recent years. Recently, Wang X et al. reported that miRNA-485-5p attenuates breast cancer cells' migratory and invasive abilities by silencing MUC1 27. Another study showed that miR-485-5p could downregulate ovarian cancer progression by targeting SRC 28. In the present study, we observed that downregulation of miR-485-5p could suppress the expression of IGF2BP3 and promote the proliferation and metastasis ability of FaDu cells. Dual-luciferase assays also determined the direct relationship of IGF2BP3 and miR-485-5p. These results demonstrated that miR-485-5p inhibits HSCC cancer cell proliferation and metastasis via IGF2BP3.

Studies have shown that lncRNAs are significantly correlated with adjacent protein-coding genes and may influence protein-coding gene expression at the transcriptional and post-transcriptional levels 32. Thus, we screened out the only upregulated protein-coding gene within 20 kb of linc01224 in our RNA sequencing data, IGF2BP3. Moreover, the expression of IGF2BP3 was negatively related to the overall survival of HSCC.

Insulin-like growth factor 2 mRNA binding proteins (IGF2BPs), including IGF2BP1 (IMP1), IGF2BP2 (IMP2), and IGF2BP3 (IMP3), can suppress translation in late development; they include several KH domains that are fundamental in RNA binding, and they are also known to be involved in RNA chemical and biological processes 33,34. IGF2BP3 is expressed in a time-dependent manner in fetal tissues but not in mature tissues 35,36. Additionally, IGF2BP3 upregulation has been observed in many cancer types 37-41, supporting tumor growth and metastasis. For example, Li et al. observed that IGF2BP3 stimulated hepatocellular carcinoma cell chemoresistance and that IGF2BP3 expression correlated with poor prognosis 42. Hsu et al. achieved similar conclusions regarding ovarian cancer as well 43. Moreover, Lochhead et al. suggested that IGF2BP3-positive colorectal cancers may have more aggressive biological behavior than IGF2BP3-negative counterparts 44. Our findings also indicated that IGF2BP3 expression is significantly higher in HSCC tissues than in normal tissues and is positively related to poor prognosis. The expression of IGF2BP3 at the mRNA and protein levels was attenuated by knockdown of linc01224, which indicated that downregulation of linc01224 inhibited tumor-promoting activity in HSCC by repressing IGF2BP3 protein synthesis. Thus, we recognized that IGF2BP3 is an oncofetal protein, and IGF2BP3 may be an emerging cancer biomarker.

Based on these findings, we suggest that linc01224 increases IGF2BP3 expression to promote invasion and migration and inhibit HSCC cell apoptosis by binding to miR-485-5p. These findings may shed light on the treatment of HSCC. However, future studies should explore the mechanisms of linc01224's oncogenic activities. Additionally, we need to explore which pathways, if any, linc01224 takes part in and explore and expand the experiments to more available HSCC cell lines.

In summary, our research is the first to provide evidence that linc01224 acts as a novel tumor promoter through the miR-485-5p/IGF2BP3 axis in HSCC. It implies that linc01224 may serve as a biomarker and could be a potentially helpful target for inhibiting hypopharyngeal tumorigenesis and progression.

## Figures and Tables

**Table 1:** Correlation between clinicopathologic characteristics and IGF2BP3 expression in HSCC

Characteristic		n=28	IGF2BP3		P
			Low expression	High expression	
Age (y)		28	59.07±6.53	56.92±5.93	0.37
Tumor size (cm^3^)					
	<10	19	10	9	0.21
	≥10	9	4	5	
T stage					
	1-2	5	4	1	0.33
	3-4	23	10	13	
N stage					
	0	10	5	5	0.86
	≥1	18	11	7	
M stage					
	0	7	4	3	0.66
	1	21	10	11	

^*^p<0.05; TNM: tumor-node-metastasis.

## References

[1] Hong YM, Gan WG, Xu ZH (2014). Significance of the expression of integrin β1, VEGF and MVD in hypopharyngeal squamous cell carcinoma. Genet Mol Res.

[2] Chen W, Zheng R, Baade PD (2016). Cancer statistics in China, 2015: Cancer Statistics in China, 2015. CA Cancer J Clin.

[3] Vengaloor Thomas T, Nittala M, Bhanat E (2020). Management of Advanced-stage Hypopharyngeal Carcinoma: 25-Year Experience from a Tertiary Care Medical Center. Cureus.

[4] Siegel RL, Miller KD, Jemal A (2020). Cancer statistics, 2020. CA Cancer J Clin.

[5] Dai W, Jin X, Jiang B (2020). Elevated O-GlcNAcylation Promotes Malignant Phenotypes of Hypopharyngeal Squamous Cell Carcinoma by Stabilizing Nrf2 through Regulation of the PI3K/Akt Pathway. Anticancer Agents Med Chem.

[6] Lin P, Tian P, Pang J (2020). Clinical significance of COL1A1 and COL1A2 expression levels in hypopharyngeal squamous cell carcinoma. Oncol Lett.

[7] Kim EK, Cho YA, Koh YW (2020). Prognostic implications of Fibroblast growth factor receptor 1 (FGFR1) gene amplification and protein overexpression in hypopharyngeal and laryngeal squamous cell carcinoma. BMC Cancer.

[8] Qian Y, Liu D, Cao S (2017). Upregulation of the long noncoding RNA UCA1 affects the proliferation, invasion, and survival of hypopharyngeal carcinoma. Mol Cancer.

[9] Lin Z, Lai S, He X (2017). Decreased long non-coding RNA MTM contributes to gastric cancer cell migration and invasion via modulating MT1F. Oncotarget.

[10] Xu Q, Deng F, Qin Y (2016). Long non-coding RNA regulation of epithelial-mesenchymal transition in cancer metastasis. Cell Death Dis.

[11] Hu X, Feng Y, Zhang D (2014). A Functional Genomic Approach Identifies FAL1 as an Oncogenic Long Noncoding RNA that Associates with BMI1 and Represses p21 Expression in Cancer. Cancer Cell.

[12] Teschendorff AE, Lee S-H, Jones A (2015). HOTAIR and its surrogate DNA methylation signature indicate carboplatin resistance in ovarian cancer. Genome Med.

[13] Wei L-H, Guo JU (2020). Coding functions of 'noncoding' RNAs. Science.

[14] Zhou J, Li M, Yu W (2016). AB209630, a long non-coding RNA decreased expression in hypopharyngeal squamous cell carcinoma, influences proliferation, invasion, metastasis, and survival. Oncotarget.

[15] Zhou J, Li W, Jin T (2015). Gene microarray analysis of lncRNA and mRNA expression profiles in patients with hypopharyngeal squamous cell carcinoma. Int J Clin Exp Med.

[16] Wu H, Yu D, Wu M (2018). Long non-coding RNA LOC541471: A novel prognostic biomarker for head and neck squamous cell carcinoma. Oncol Lett.

[17] Gong D, Feng P-C, Ke X-F (2020). Silencing Long Non-coding RNA LINC01224 Inhibits Hepatocellular Carcinoma Progression via MicroRNA-330-5p-Induced Inhibition of CHEK1. Mol Ther - Nucleic Acids.

[18] Li H, Gao C, Liu L (2019). 7-lncRNA Assessment Model for Monitoring and Prognosis of Breast Cancer Patients: Based on Cox Regression and Co-expression Analysis. Front Oncol.

[19] Zhang G (2018). LncRNA MT1JP functions as a ceRNA in regulating FBXW7 through competitively binding to miR-92a-3p in gastric cancer. Mol Cancer.

[20] Yu Y, Gao F, He Q (2020). lncRNA UCA1 Functions as a ceRNA to Promote Prostate Cancer Progression via Sponging miR143. Mol Ther - Nucleic Acids.

[21] Xing S, Zhang Y, Zhang J (2020). LINC01224 Exhibits Cancer-Promoting Activity in Epithelial Ovarian Cancer Through microRNA-485-5p-Mediated PAK4 Upregulation. OncoTargets Ther.

[22] Trapnell C, Williams BA, Pertea G (2010). Transcript assembly and quantification by RNA-Seq reveals unannotated transcripts and isoform switching during cell differentiation. Nat Biotechnol.

[23] He J, Huang B, Zhang K (2020). Long non-coding RNA in cervical cancer: From biology to therapeutic opportunity. Biomed Pharmacother.

[24] Li X, Xu F, Meng Q (2020). Long noncoding RNA DLEU2 predicts a poor prognosis and enhances malignant properties in laryngeal squamous cell carcinoma through the miR-30c-5p/PIK3CD/Akt axis. Cell Death Dis.

[25] Li N, Yang G, Luo L (2020). lncRNA *THAP9-AS1* Promotes Pancreatic Ductal Adenocarcinoma Growth and Leads to a Poor Clinical Outcome via Sponging miR-484 and Interacting with YAP. Clin Cancer Res.

[26] Chen J (2019). Long non-coding RNA PVT1 promotes tumor progression by regulating the miR-143/HK2 axis in gallbladder cancer. Mol Cancer.

[27] Wang X, Zhou X, Zeng F (2020). miR-485-5p inhibits the progression of breast cancer cells by negatively regulating MUC1. Breast Cancer Tokyo Jpn.

[28] Yang Y, Liu J, Qian X (2020). miR-485-5p improves the progression of ovarian cancer by targeting SRC *in vitro* and *in vivo*. Neoplasma.

[29] Liu Q, Wang Z, Zhou X (2020). miR-485-5p/HSP90 axis blocks Akt1 phosphorylation to suppress osteosarcoma cell proliferation and migration via PI3K/AKT pathway. J Physiol Biochem.

[30] Chen Z, Li Q, Wang S (2015). miR-485-5p inhibits bladder cancer metastasis by targeting HMGA2. Int J Mol Med.

[31] Duan J, Zhang H, Li S (2017). The role of miR-485-5p/NUDT1 axis in gastric cancer. Cancer Cell Int.

[32] Kornienko AE, Guenzl PM, Barlow DP (2013). Gene regulation by the act of long non-coding RNA transcription. BMC Biol.

[33] Nielsen J, Christiansen J, Lykke-Andersen J (1999). A Family of Insulin-Like Growth Factor II mRNA-Binding Proteins Represses Translation in Late Development. Mol Cell Biol.

[34] Samuels TJ, Järvelin AI, Ish-Horowicz D (2020). Imp/IGF2BP levels modulate individual neural stem cell growth and division through myc mRNA stability. eLife.

[35] Mueller-Pillasch F, Pohl B, Wilda M (1999). Expression of the highly conserved RNA binding protein KOC in embryogenesis. Mech Dev.

[36] Mancarella C, Scotlandi K (2020). IGF2BP3 From Physiology to Cancer: Novel Discoveries, Unsolved Issues, and Future Perspectives. Front Cell Dev Biol.

[37] Findeis-Hosey JJ, Xu H (2011). The use of insulin like-growth factor II messenger RNA binding protein-3 in diagnostic pathology. Hum Pathol.

[38] Okabayshi M, Kataoka TR, Oji M (2020). IGF2BP3 (IMP3) expression in angiosarcoma, epithelioid hemangioendothelioma, and benign vascular lesions. Diagn Pathol.

[39] Zhou Y, Huang T, Siu HL (2017). IGF2BP3 functions as a potential oncogene and is a crucial target of miR-34a in gastric carcinogenesis. Mol Cancer.

[40] Jiang W, Cheng X, Wang T (2020). LINC00467 promotes cell proliferation and metastasis by binding with IGF2BP3 to enhance the mRNA stability of TRAF5 in hepatocellular carcinoma. J Gene Med.

[41] Liu H, Zeng Z, Afsharpad M (2019). Overexpression of IGF2BP3 as a Potential Oncogene in Ovarian Clear Cell Carcinoma. Front Oncol.

[42] Li M, Zhang L, Ge C (2015). An isocorydine derivative (d-ICD) inhibits drug resistance by downregulating IGF2BP3 expression in hepatocellular carcinoma. Oncotarget.

[43] Hsu K-F, Shen M-R, Huang Y-F (2015). Overexpression of the RNA-binding proteins Lin28B and IGF2BP3 (IMP3) is associated with chemoresistance and poor disease outcome in ovarian cancer. Br J Cancer.

[44] Lochhead P, Imamura Y, Morikawa T (2012). Insulin-like growth factor 2 messenger RNA binding protein 3 (IGF2BP3) is a marker of unfavourable prognosis in colorectal cancer. Eur J Cancer.

